# Musician's dystonia: an opinion on novel treatment strategies

**DOI:** 10.3389/fnins.2024.1393767

**Published:** 2024-04-08

**Authors:** Joy Grifoni, Valeria Crispiatico, Anna Castagna, Angelo Quartarone, Rosa Maria Converti, Marina Ramella, Giuseppe Granata, Riccardo Di Iorio, Alfredo Brancucci, Gabriela Bevacqua, Marco Pagani, Teresa L'Abbate, Karolina Armonaite, Luca Paulon, Franca Tecchio

**Affiliations:** ^1^Uninettuno International University, Rome, Italy; ^2^Laboratory of Electrophysiology for Translational neuroScience LET'S, Institute of Cognitive Sciences and Technologies ISTC, Consiglio Nazionale delle Ricerche CNR, Rome, Italy; ^3^Milano Bicocca University, Milan, Italy; ^4^IRCCS Fondazione Don Carlo Gnocchi, Milan, Italy; ^5^IRCCS Bonino Pulejo, Messina, Italy; ^6^Neurology Unit, Fondazione Policlinico Universitario Agostino Gemelli IRCCS, Rome, Italy; ^7^Dipartimento di Scienze Motorie, Umane e della Salute, Università di Roma “Foro Italico”, Rome, Italy; ^8^Studio Psyche Neuroscienze, Rome, Italy; ^9^Independent Researcher, Rome, Italy

**Keywords:** task-specific musician's dystonia (MD), psycho-neurophysiology, identity trauma, feedback-synchrony-plasticity FeeSyCy principle, sensory-motor integration, multi sensorymotor rehabilitation (EMDR+^®^), transcranial direct current stimulation (tDCS)

## Introduction

In this opinion paper, we contribute our perspective on the potential of recent findings from relevant pathophysiological contexts to indicate therapeutic approaches in musicians' dystonia (MD). This neurological condition is mostly painless and highly task-specific, with symptoms only occurring when trying to play the instrument, and includes two most common forms: hand, and embouchure dystonia. We hope to encourage constructive discussion about a multidimensional strategy to alleviate this disabling symptom. In the realm of neuroscientific discourse, our viewpoint on this condition is framed as an opinion that emphasizes the imperative of *early and precise diagnosis*, alongside the *personalized* application of therapeutic modalities. This opinion arises from a synthesis of empirical evidence and theoretical insights, advocating for a nuanced approach that extends beyond mere symptom management. We contend that the efficacy of interventions hinges upon a *comprehensive understanding* of the multifaceted nature of MD, encompassing its neurobiological underpinnings and its psychosocial ramifications.

This opinion underscores the necessity of employing a diverse array of intervention strategies: this encompasses interventions targeting the sensorimotor system, such as physiotherapy, via interventions addressing the subcortical domains implicated in memory consolidation, identity integration, and emotion regulation, a paradigm that acknowledges the intricate interplay between neural circuits and psychological processes. By embracing this comprehensive perspective, we aim to foster symptom alleviation, cultivating resilience, adaptive coping mechanisms, and enhanced quality of life for individuals grappling with this challenging neurological condition.

## Pathophysiological aspects

Research into task-specific focal dystonia in musicians has revealed that its primary pathophysiological mechanism involves impaired sensorimotor integration within circuits connecting the basal ganglia, cerebellum, and sensorimotor cortices (Rosenkranz et al., 2005). This impairment is characterized by abnormalities in cortical somatosensory processing, disrupted sensorimotor integration, and maladaptive sensory-motor plasticity (Quartarone and Hallett, 2013).

As previously presented (Salimpoor et al., 2015; Tecchio et al., 2020; Persichilli et al., 2022; Grifoni et al., 2023), the brain is a network composed of groups of neurons that work together. These ensembles of neurons collaborate enabling the body-brain to kengage with the environment. We introduced the term “FeeSyCy” which stands for feedback, synchrony, and plasticity. It refers to a triadic principle that governs the coordination of the body-brain network interaction with the environment. The brain receives feedback from sensory receptors, such as visual, auditory, and proprioceptive receptors (Flanagan and Johansson, 2003; Fink et al., 2014). This feedback is related to the actions that the person is currently performing, and it depends on the goals it is trying to achieve (Friston, 2018). The receipt of feedback is associated with the synchronization or desynchronization of electrical activities in different regions of the brain. This synchronization determines whether the planned action should continue as intended or if it needs modification through plastic changes in the brain's neural connections to adapt to changing environmental demands (Wolpert et al., 2011).

Historically, the impairment of motor control in MD parallels the alteration of the somatosensory representation of the dystonic body region observed in task-specific dystonia in animal models and in musicians (Candia et al., 2003). Recent data lend weight to an alternative hypothesis that task-specific dystonia is due to a higher-order disruption of skill encoding (Doll-Lee et al., 2023; Furuya and Oku, 2023; Sadnicka et al., 2023). This evidence supports therapeutic interventions aimed at the specific skill involved and its control network, supporting the interruption of ingrained motor behaviors by injecting variability into movement repetitions (Sadnicka and Rosset-Llobet, 2019).

Musicians face significant psychological stress or identity trauma due to intense early training, public performance demands, and direct abuse or dysfunctional social interactions within both the educational and broader social environments (Ioannou et al., 2016; Détári and Egermann, 2022). This emotional toll, combined with high expectations for perfection and personal sacrifice, can lead to emotional trauma and mental health disorders in musicians (Schoeb and Zosso, 2012; Ioannou et al., 2016), contributing to the development of MD (Altenmüller and Jabusch, 2009; Alpheis et al., 2022). Considering that many musicians with intense practice and performance schedules cope with the musical landscape without serious issues, is increasing awareness of how early adverse traumatic experiences and negative upbringing can significantly impact future MD symptomatology (Alpheis et al., 2023).

## Point of view for discussion of a new multidimensional therapeutic strategy

Drawing insights from interconnected pathophysiological conditions, we advocate for a comprehensive, multimodal therapeutic approach that recognizes the multifaceted nature of the condition. This perspective stems from acknowledging the various factors contributing to MD, which encompass physiological, psychological, and social dimensions. At the core of our viewpoint lies the belief that effective treatment requires collaborative engagement between patients and therapists, supported by a multidisciplinary team comprising healthcare professionals with expertise in neurology, psychology, rehabilitation, and related fields.

This multifaceted approach aims to address the symptomatic manifestations of MD while promoting resilience, empowering patients to regain agency in their interaction with the environment, and enhancing overall wellbeing for individuals grappling with MD.

### Personalized physiotherapy

The Sensory-Motor Retuning strategy focuses on retraining the brain's sensory and motor pathways to alleviate symptoms of dystonia. Introduced by Candia et al. (1999), it involves immobilizing non-dystonic fingers with splints while the dystonic finger undergoes repetitive exercises. By doing so, it aims to induce neuroplastic changes in the brain, promoting sensorimotor integration and normalizing aberrant neural activity associated with task-specific dystonia. Complementing this approach, modified Graded Motor Imagery (mGMI), as proposed by Ramella et al. (2021) in line with Butler et al. (2021), incorporates visual feedback training and mental imagery exercises, acknowledging the multidimensional feedback reprocessing required to recover from MD. Considering the relevance of multi-modal feedback in motor control, this strategy is designed to train visual feedback by performing visual exercises to reprocess the visual information from the dystonic hand, training movement without execution to alleviate dystonia-induced suffering, and performing the same motor exercises with both hands receiving visual feedback while looking at the non-dystonic hand together with its mirrored image replacing that of the dystonic hand (mirror box). To focus attention on the specific alterations of the musician with task-specific dystonia, it is appropriate to use adequate scales or targeted interviews to account for the prevalence of feedback processing disorders as a critical counterpart of the expression of symptomatology. In current scientific literature there are no rehabilitation guidelines and/or studies that standardize treatments in terms of modality, duration and/or intensity. Recent systematic reviews (Enke and Poskey, 2018; Chiaramonte and Vecchio, 2021) have highlighted that the most used treatments are sensorimotor ri-education, task-specific motor training, and Graded Motor Imagery.

### Personalized psychotherapy

Recognition and intervention regarding *emotional trauma* constitute a fundamental aspect of recent therapeutic approaches in MD (Détári and Egermann, 2022). To provide material for discussion, we present an example standing on Eye Movement Desensitization and Reprocessing (EMDR), which the WHO recognizes as an elective trauma treatment (World Health Organization, 2013). EMDR is a therapy aimed at reprocessing traumatic memories to integrate them into existing memory networks in a less distressing manner. It combines bilateral sensory stimulation and cognitive processing to mimic REM sleep, aiding memory consolidation and processing.

**Figure 1:**
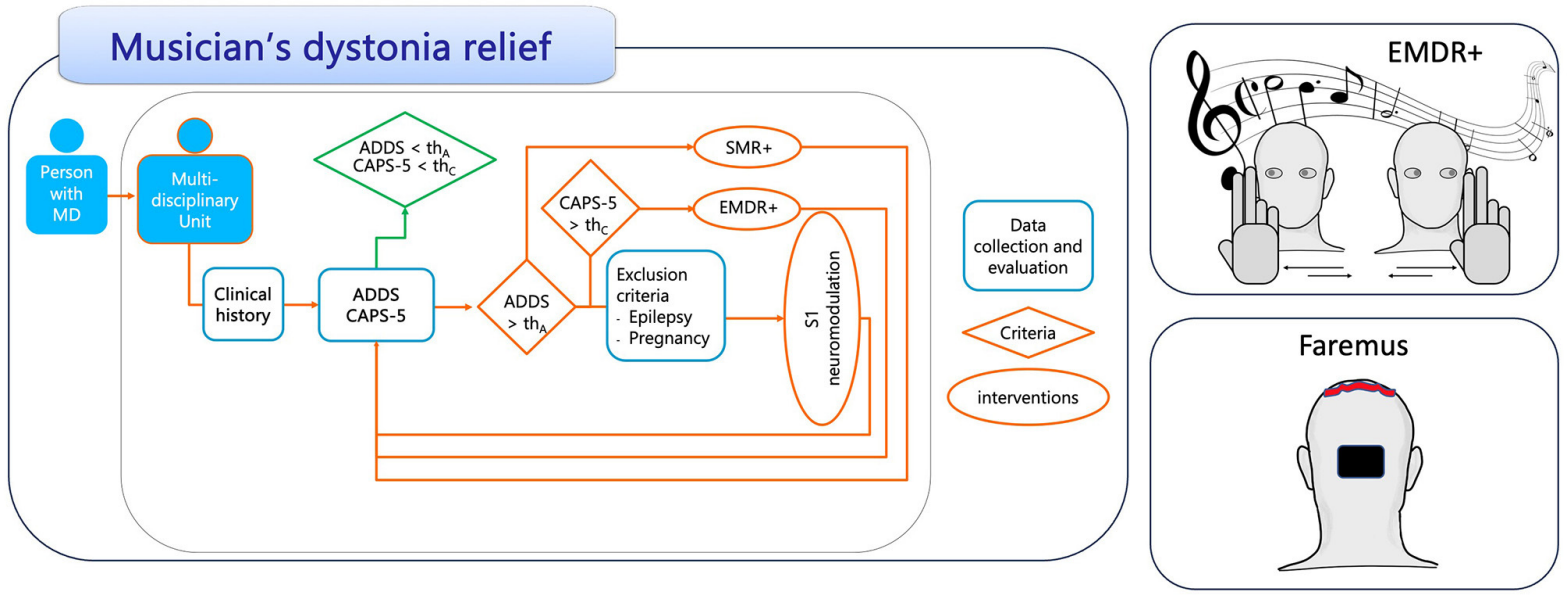
Personalized multidimensional treatment to relieve dystonia in musicians. Schematic representation of the therapeutic strategy algorithm. As an example, we have introduced scales for inclusion and monitoring: ADDS, Arm Dystonia Disability Scale; CAPS-5, Clinician-Administered PTSD Scale; “th,” severity threshold related to each scale; SMR+, SensoryMotor Retuning personalized by modified Graded Motor Imagery; EMDR+^®^, EMDR with auditory personalization; S1 neuromodulation, tDCS with the anode on the bilateral somatosensory representation of the whole-body and a double-sized electrode cathode on occipital area.

While comprehension of EMDR mechanisms of action is ongoing, it is ensured that it exploits and supports the orienting reflex, working memory saturation, and slow-wave-sleep cerebral phenomena (Persichilli et al., 2022). Therapists collaborate with the patient to identify and target specific distressing memories or aspects of trauma, promoting meaningful resolution and symptom relief. EMDR structures, in eight phases, the therapeutic pathway monitoring the person's state by devoted scales, the Subjective Units of Disturbance and the Validity of Cognition, and utilizes bilateral alternate stimuli via eye movements led by the therapist (Figure 1) as a key part of the desensitization phase, when detachment from the traumatic event occurs.

Taking advantage of the therapeutic capacity of music (Pant et al., 2022), the *EMDR*+^®^ strategy (Grifoni et al., 2023) has recently been proposed, showing promising feasibility, safety, acceptance, and evident efficacy in individuals suffering from trauma-induced psychological symptoms. EMDR+^®^ introduces music within the therapeutic process, capitalizing on its *motivational potential* and *emotional impact* (Salimpoor et al., 2015).

EMDR+^®^ therapy, anchored in the *reward-based* attributes of music and bilateral sound stimulation, offers a unique and potentially transformative strategy for tackling the traumatic etiology of task-specific focal dystonia in musicians. It enriches the EMDR protocol with sound stimulation by administering also neutral sounds synchronized with the guided bilateral alternating stimulation of the gaze, thus engaging multiple senses simultaneously (Silva et al., 2015).

For musicians, music transcends mere artistry and constitutes a vocational ground for social relationships (Menon and Levitin, 2005). Via EMDR+^®^, music assumes the role of a potent motivator (Salimpoor et al., 2015) and an integral component of the healing process, addressing a critical dimension of overloading emotional trauma resolution. This integration (Dresel et al., 2014) resonates with their personal preferences (Rentfrow and Gosling, 2003). Selection based on the patients' *predisposition* and personal *tastes* relates to two main components of the therapeutic path. A *key song* supports the evocative potential for trauma-related memory assessment and a *reward song* for consolidating positive elaboration.

To obtain the clinical profile of the patient's psycho-emotional percept at the beginning and at the end of the therapeutic EMDR+^®^ course, the therapist administers EMDR traditional scales but also proper scales of anxiety and stress, like the Clinician-Administered PTSD Scale (CAPS-5), Hamilton Anxiety Rating Scale (HARS), BDI, PHQ (Weathers et al., 2001).

### Personalized neuromodulation

Overcoming the sensorimotor integration deficit typical of MD involves multiple levels of reconstruction of motor control processing (Sadnicka et al., 2018). The intracerebral short circuits typical of stereotyped movement and trauma-related effects can be relieved through neuromodulation, the family of techniques that allow the excitability of neuronal targets to be modified, thus modifying exchanges with other connected areas (Lefaucheur et al., 2017). Although the evidence for the effectiveness of neuromodulation for MD is still weak and inconsistent (Menon et al., 2023), we pose as an object of discussion that the experience of the Authors in mitigating fatigue may also be an enriching strategy relevant for alleviating MD, since the two conditions share relevant pathophysiological mechanisms. Specifically, data gathered investigating the neural pathophysiological traits of task-specific dystonias reported that a musician's dystonia was shaped by alterations in primary and secondary sensorimotor cortices pointing to impairments of sensorimotor guidance within executive control (Bianchi et al., 2019). The data on the involvement of the sensorimotor network and on the modifications due to the non-invasive brain stimulation developed to relieve fatigue (S1 neuromodulation) speak in favor of a correct rebalancing of the previously interrupted integrative relationship within the complex control circuit of the hand induced by this intervention (Porcaro et al., 2019; Padalino et al., 2021). S1 neuromodulation involves transcranial direct current stimulation (tDCS) for 5 days, for 15 min per day, targeted via the anode to the bilateral whole-body primary somatosensory representation with the cathode over the occipital region using a double area electrode to reduce effects in this region to a minimum. The logic of this intervention is evidence for the physiological foundation of symptoms including postcentral hypoactivity and precentral hyperactivity. Among tDCS interventions to mitigate MD, the most promising targeted central-parietal areas (Rosset-Llobet et al., 2015). We note that in that intervention, the targets of neuromodulation were left vs. right primary sensorimotor homologs, whereas bilateral alterations are typically observed in task-specific focal dystonia (Melgari et al., 2013; Dresel et al., 2014), suggesting the appropriateness of a bilateral intervention. On this basis, we believe that the proposed S1 neuromodulation (Tecchio et al., 2014; Cancelli et al., 2018) can offer a promising therapeutic enrichment, inducing bilateral rather than unilateral facilitation, and focusing the facilitatory effects on somatosensory rather than sensorimotor areas supporting the physiological sensorimotor integration processing (Porcaro et al., 2019; Padalino et al., 2021; Bertoli et al., 2023).

## Representation of the pathway of a multidimensional therapeutic strategy

As detailed above for each therapeutic dimension, we present here, for discussion, a personalized therapeutic strategy (Figure 1). Individuals with MD can seek assistance from a multidisciplinary team of experts. If a proper scale indicates severity above the threshold, the therapeutic strategy is initiated based on the individual's profile. For individuals exhibiting more severe signs of traumatic effects, personalized psychotherapy is recommended. Neuromodulation may be considered if SMR+ and EMDR+^®^ fail to yield the expected clinical improvements. It should be noted that quantifying both the inclusion criterion at the beginning of therapy and the evolution of individuals with MD in response to the therapeutic process using standardized scales is crucial. In the schematic algorithmic representation, we have maintained the same scale for enrollment and monitoring, but future study designs should explore whether the enrollment scale also exhibits sufficient sensitivity to monitor the evolution of individuals with MD.

In particular, it is possible to provide remote treatments for individuals who may face obstacles in accessing traditional in-person treatment modalities and to facilitate adherence to repeated sessions, when necessary. In particular, EMDR+^®^ is already applied online (unpublished data), S1 neuromodulation has been established at home with efficacy comparable to use in the clinic (Tecchio et al., 2022).

## Discussion

In accordance with the multidimensional understanding and treatment of MD (Détári, 2023), the presented approach stimulates discussion to renew the relationship between dystonic musicians and rehabilitators. The two counterparts, involving physical activity and psychotherapeutic treatment, use body-oriented methods to optimize playing behaviors, as suggested by Détári (2023). The therapeutic approach aims to consciously channel the focus of attention to guide physical retraining exercises and establish new habits aiming to emphasize the positive aspects currently available, concentrating thoughts on the communicative contents rather than on the performance of the musical piece. Going beyond the weak suggestions coming from NIBS in task-specific focal dystonia (Menon et al., 2023), our novel view suggests that supporting physical and psychotherapeutic detailed personalization can make use of a possible neuromodulation supporting protocol (Gianni et al., 2021) to facilitate intracerebral communication by overcoming sensory-motor integration disorders.

### Limitations of this opinion

Everything that has been proposed requires experimental testing and multicenter randomized controlled trials to evaluate the effectiveness in alleviating the symptoms of MD. These trials will be personalized to treat each patient's unique symptoms and the specific ways that *trauma* affects them. Once we see positive results, we'll further adjust the treatment to better suit individual characteristics like the patient's age, the musical instrument they play, and how advanced their condition is. Our method can be delivered also in remotely, which makes it easier to adhere to the pathway and to extend to more patients. An important part of our strategy is to keep fine-tuning the therapy based on the patient's ongoing feedback and their progress. This ensures that the treatment continues to meet their evolving needs, which is crucial for achieving the best possible results.

### Strengths of this opinion

We propose here an informed discussion based on the known mechanisms of musicians' dystonia and on therapeutic strategies that we expect to alleviate the dysfunction in MD by capitalizing on interventions capable of alleviating the alterations common to other clinical conditions.

## Author contributions

FT: Conceptualization, Supervision, Visualization, Writing – original draft, Writing – review & editing. JG: Conceptualization, Supervision, Writing – original draft, Writing – review & editing, Data curation, Investigation, Project administration, Visualization. VC: Conceptualization, Data curation, Formal analysis, Investigation, Methodology, Project administration, Resources, Supervision, Validation, Visualization, Writing – original draft, Writing – review & editing. AC: Supervision, Validation, Visualization, Writing – review & editing. AQ: Conceptualization, Supervision, Validation, Visualization, Writing – review & editing. RC: Conceptualization, Supervision, Validation, Visualization, Writing – review & editing. MR: Conceptualization, Supervision, Validation, Visualization, Writing – review & editing. GG: Supervision, Validation, Visualization, Writing – review & editing. RD: Supervision, Validation, Visualization, Writing – review & editing. AB: Conceptualization, Supervision, Validation, Visualization, Writing – review & editing. GB: Validation, Visualization, Writing – review & editing. MP: Supervision, Validation, Visualization, Writing – review & editing. TL'A: Data curation, Validation, Visualization, Writing – review & editing. KA: Validation, Visualization, Writing – review & editing. LP: Software, Supervision, Validation, Visualization, Writing – review & editing.
